# IMPLICATION OF PAIN ON MOBILITY, FALLS, AND PSYCHOLOGICAL OUTCOMES FOR PEOPLE LIVING WITH DEMENTIA

**DOI:** 10.1093/geroni/igac059.1588

**Published:** 2022-12-20

**Authors:** Annalisa Na, Leslie McClure, Rose Ann DiMaria-Ghalili, Laura Gitlin

**Affiliations:** Drexel University, Philadelphia, Pennsylvania, United States; Drexel University, Philadelphia, Pennsylvania, United States; Drexel University, College of Nursing and Health Professions, Philadelphia, Pennsylvania, United States; Drexel University, Philadelphia, Pennsylvania, United States

## Abstract

Dementia and pain can challenge mobility, increase fall risk, and result in psychological consequences; however, the extent of these relationships among community-dwelling people living with dementia (PLWD) is unclear. Using the National Health and Aging Trends Study, we examined 9,974 community-dwelling older adults (female=57%, age>85yrs=18%) for pain prevalence, mobility (i.e., Short Physical Performance Battery [SPPB]), falls, and psychological outcomes (i.e., depression and anxiety) using four groups with and without dementia (+PLWD/-PLWD) and pain (+Pain/-Pain). Groups were compared at baseline with ANOVA and chi-square and over time with repeated-measures ANOVA models. Pain (+PLWD= 56.8%; -PLWD=49.8%) most commonly affected the low-back and knee. Group differences were significant for baseline mobility, falls, and psychosocial outcomes, p<0.01, but not SPPB over time. Therefore, pain is common, especially at low back and knee, and associated with health and function among PLWD, indicating a need for early and effective interventions that can preserve quality of life.

